# 620 The Effectiveness of Aromatherapy in an Urban-Based, Safety-Net Hospital on Pain and anxiety

**DOI:** 10.1093/jbcr/irac012.248

**Published:** 2022-03-23

**Authors:** Rebecca Coffey, Justin Buchert, Alina Ruiz, Jillian Alcorn, June Van Hoose

**Affiliations:** Parkland Health Hospital System, Dallas, Texas; Parkland Health and Hospital System, Dallas, Texas; Parkland Health and Hospital System, garland, Texas; Parkland Hospital, Lancaster, Texas; Parkland Health and Hospital System, Carrollton, Texas

## Abstract

**Introduction:**

Essential oils have been used for centuries for their calming properties. This alternative medicine therapy is gaining popularity in the inpatient setting for symptom management including pain, anxiety, nausea, and insomnia. The purpose of this project is to evaluate the effectiveness of aromatherapy complement as an alternative and/or treatment for pain and anxiety ion the acute care setting at on Health and Hospital System, The target population for this study was patient who were receiving care in the Surgical Services inpatient units including burn patients.

**Methods:**

This study included all surgical patients admitted to one hospital and this is an analysis of the burn cohort enrolled thus far in this study. All adult burn patients that met inclusion criteria were included to evaluate the impact of aromatherapy on the administration of pain and anxiety medications (PANX). After introducing the patient to the study and obtaining informed consent a Lavender-Sandalwood scented aromatherapy sticker was placed on the patients' gown. The aroma last from the sticker last for twelve hours and could be replaced upon patient request. The stickers were used for a total of three days. Survey data was collected to describe the experience with the sticker as well as PANX medication administration and demographic information. Pain scores were recorded for each patient before PANX administration within the 72 hour time period after surgery or burn wound care procedures.

**Results:**

To date a total of fourteen patients were enrolled in the study mean age was 44 years old, 71% of the patients were male and the mean TBSA was 5%.Two patients withdrew one because the scent gave them a headache and one because there were no study logs completed. Each of the twelve patients used a total of 6 stickers over the 3 days of the intervention. Prior to the start of the study the average pain score was 8.6 (range 7-10). On day one of the intervention the mean pain score was 4.75 (range 3-8), day two 4.25 (range 2-8), and on day three 4.3 (range 0 - 9). Post intervention pain scores were 5.75 (range 0-9). Comparison of the pain scores pre and post intervention were statistically significant t= -2.904(95% CI = -4.85 to -0.81) p = 0.0082. Of note patients reported that sleep was improved while using the aromatherapy sticker.

**Conclusions:**

Aromatherapy may provide a natural alternative to reduce opioid pain medications and improve sleep in the burn patient thereby improving long term outcomes.

